# P95 - Cow’s milk allergy with tolerance to sterilised cow’s milk. A case report

**DOI:** 10.1186/2045-7022-4-S1-P150

**Published:** 2014-02-28

**Authors:** Liseth Villafana, Soledad Terrados, Amalia Ledesma

**Affiliations:** 1 Ramón y Cajal Hospital, Madrid, Spain; 2 ALK- Albelló Spain, Madrid, Spain

## Introduction

Allergy to cow's milk (CM) proteins has as major allergens caseins (60%), β-lactoglobulin (9%) and α-lactalbumin (4%). Thermal processes for milk industrialization include: pasteurization (low: 63 ° C for 30 min or High: 72 ° C for 15 seconds), UHT heating (145 ° C for 1-2s) and sterilization (115 ° C for 15 min).It’s accepted that these processes denature milk proteins in different proportions without having an effect on antigenicity or allergenicity.

## Case report

A 2 years old boy, exclusively breastfed up to 3 months and then fed with adapted infant formula with good tolerance until 18 months. Referred by his pediatrician with suspected CM allergy because of immediate perioral erythema and pruritus after consumption of milk and some dairy products (yoghurt) during the 2 last months. However, he tolerates a sterilized cow’s milk and cheese. He hadn’t got any concomitant systemic symptoms like dyspnea, vomiting, or diarrhea. Skin test were performed: CM (5mm), α-lactalbumin (24mm), β-lactoglobulin (11mm), casein (20mm), sterilized milk (SM) (3mm), pasteurized milk(PM) (11mm), UHT milk (UM)(9mm). Histamine (6mm) and Saline (0mm). Provocation test with yoghurt: Positive (cervicofacial urticaria).

SM, PM and UM extracts were analysed using SDS-PAGE and immunoblooting with patient’s serum and with a pool of sera from CM allergic patients. Patient's serum recognized a band of about 13 kDa (molecular weight described for the alpha-lactalbumin( 14 kDa) in the UM and PM, but not in sterilized milk.

## Comments

The patient has a primary sensitization to α-lactalbumin protein fraction of cow´s milk. According to our study, this fraction is not recognized by the patient in sterilized milk so we conclude that, in contrast to the accepted, this method of treatment do alters the structure of at least this fraction of the milk which decreasing its allergenicity. We cannot *demonstrate* this with other industrial **processes.**

